# Somatic and cognitive-affective depressive symptoms among patients with heart disease: differences by sex and age

**DOI:** 10.1590/0104-1169.0287.2544

**Published:** 2015

**Authors:** Carina Aparecida Marosti Dessotte, Fernanda Souza Silva, Rejane Kiyomi Furuya, Marcia Aparecida Ciol, Jeanne Marie Hoffman, Rosana Aparecida Spadoti Dantas

**Affiliations:** 1 PhD, Professor, Escola de Enfermagem de Ribeirão Preto, Universidade de São Paulo, WHO Collaborating Centre for Nursing Research Development, Ribeirão Preto, SP, Brazil; 2 Doctoral student, Escola de Enfermagem de Ribeirão Preto, Universidade de São Paulo, WHO Collaborating Centre for Nursing Research Development, Ribeirão Preto, SP, Brazil. Scholarship holder from Coordenação de Aperfeiçoamento de Pessoal de Nível Superior (CAPES), Brazil; 3 Post-doctoral fellow, Escola de Enfermagem de Ribeirão Preto, Universidade de São Paulo, WHO Collaborating Centre for Nursing Research Development, Ribeirão Preto, SP, Brazil. Scholarship holder from Fundação de Amparo à Pesquisa do Estado de São Paulo (FAPESP), Brazil; 4 PhD, Associate Professor, Department of Rehabilitation Medicine, School of Medicine, University of Washington, Seattle, WA, United States; 5 PhD, Associate Professor, Escola de Enfermagem de Ribeirão Preto, Universidade de São Paulo, WHO Collaborating Centre for Nursing Research Development, Ribeirão Preto, SP, Brazil

**Keywords:** Depression, Cardiovascular Diseases, Sex

## Abstract

**OBJECTIVE:**

this study investigated the association of somatic and cognitive-affective symptoms with sex and age, among patients hospitalized with heart disease.

**METHOD:**

this study was a secondary analysis of two previous observational studies totaling 531 patients with heart disease, hospitalized from 2005 to 2011 in two public hospitals in Ribeirão Preto, state of São Paulo, Brazil. Somatic and cognitive-affective symptoms were assessed using the subscales of the Beck Depression Inventory - I (BDI-I).

**RESULTS:**

of 531 participants, 62.7% were male, with a mean age 57.3 years (SD= 13.0) for males and 56.2 years (SD= 12.1) for females. Analyses of variance showed an effect of sex (p<0.001 for somatic and p=0.005 for cognitive-affective symptoms), but no effect of age. Women presented with higher mean values than men in both BDI-I subscales: 7.1 (4.5) vs. 5.4 (4.3) for somatic, and 8.3 (7.9) vs. 6.7 (7.2) for cognitive-affective symptoms. There were no differences by age for somatic (p=0.84) or cognitive-affective symptoms (p=0.84).

**CONCLUSION:**

women hospitalized with heart disease had more somatic and cognitive-affective symptoms than men. We found no association of somatic and cognitive-affective symptoms with age. Future research for these patients could reveal whether these differences according to sex continue throughout the rehabilitation process.

## Introduction

Cardiomyopathies are the main cause of morbidity and mortality in the world^(^ 1 ^)^. Despite the significant advances in treatment and control of cardiomyopathies, they still represent a highly relevant health problem in the world today. The World Health Organization (WHO) estimates that the global mortality due to cardiomyopathies will increase from 17.1 million in 2004 to 23.4 million in 2030, with a larger relative increase in countries of low and medium income^(^ 1 ^)^. Emotional issues in person with cardiomyopathy have been studied, including studies of depression in patients in Brazil^(^ 2 ^-^ 3 ^)^ and other countries^(^ 4 ^-^ 5 ^)^.

Depression symptoms are present in one out of five persons with coronary artery disease (CAD) and in one out three persons with congestive heart failure (CHF). However, the majority of these cases are not recognized or treated appropriately. Depression is considered a risk factor for developing cardiovascular disease, as well as a predictor of worse outcomes among persons with established cardiomyopathy^(^ 6 ^)^. 

Age and sex are two factors that have been studied as moderators of depression symptoms in persons with cardiomyopathy. Females tend to have a two to three times higher probability of being diagnosed with major depressive disorder (MDD) than males^(^ 7 ^)^, and present higher scores than males in self-report measures of depression symptoms^(^ 2 ^,^ 8 ^)^. For age, studies have shown that there is a higher prevalence of depression symptoms among adults 35 to 50 years of age, with a decline in prevalence for 65 years and older^(^ 2 ^,^ 7 ^,^ 9 ^-^ 10 ^)^. 

Researchers have also pointed out differences between men and women diagnosed with MDD regarding perception of depressive symptoms as measured by the Beck Depression Inventory (BDI-I and BDI-II)^(^ 8 ^,^ 11 ^)^. Women tended to present higher frequency of depression symptoms than men, as well as higher rates of anxiety disorders and somatic symptoms, such as fatigue, sleep disorders, and lack of appetite^(^ 12 ^)^. Among patients with cardiomyopathy, especially the ones with CAD, the presence of anxiety has been observed in various studies^(^ 12 ^-^ 13 ^)^. Given the frequency of reports of depression as well as other symptoms associated with cardiomyopathy, it is of interest to determine whether the differences between men and women exist when examining the specific symptom clusters of depression. 

Despite the research on depression and cardiomyopathy, little has been examined on whether differences exist in the presence or absence of specific depressive symptoms in this population. Specifically, we have not found studies that investigated the role of sex and age in the presence of somatic and cognitive-affective symptoms among persons with cardiomyopathy. This study aimed to investigate the association of somatic and cognitive-affective symptoms with sex and age among patients hospitalized with heart disease in two public hospitals in the state of São Paulo, Brazil. Given the previous research, we hypothesized that women would report more symptoms in each symptom cluster than men and that those who were older who report higher levels of each symptom than those who were younger. The results will help clinicians to understand the somatic and cognitive-affective symptoms profile of patients with established cardiomyopathy, by providing information that can be used in their treatment and care, and can assist in the mental and psychosocial rehabilitation of those patients. 

## Methods

### Setting and study design 

This study is a secondary analysis of data from two observational studies, which will be described below.

### Data collection and instruments

The data for this study came from two previous studies involving patients hospitalized with heart disease in two public hospitals in the state of São Paulo, Brazil, and which collected the Beck Depression Inventory - I (BDI-I) . 

The first study was performed to culturally adapt and evaluate the psychometric properties of the Antonovsky's sense of coherence questionnaire to the Portuguese language^(^ 14 ^)^. The data was collected from a single university hospital that serves individuals through the public system of healthcare in Brazil. Individuals could be included in the study if they had a diagnosis of cardiac disease confirmed by clinical, laboratory or radiologic exam, did not have psychiatric conditions (such as dementia, clinical depression or schizophrenia), and were hospitalized for diagnostic or therapeutic purposes due to cardiomyopathies, whether the first or a repeated hospitalization for the diagnosis. Data for that study were collected in two phases: 2005 to 2007 (203 participants), and 2010 to 2011 (100 participants). 

The second study was performed to study the association between depressive symptoms and age and sex, in persons with acute coronary syndrome (including acute myocardial ischemia and unstable angina) hospitalized for their first cardiac event^(^ 2 ^)^. Data were collected from the same university hospital as the first study with additional participants from a second hospital (non-teaching institution), also within the public system of healthcare, with a similar population of patients. Individuals could participate in the study if they were being hospitalized for the first episode of unstable angina or acute myocardial infarction, and were in physical and psychological condition to answer the questionnaires. Data for that study were collected from 2006 to 2009 (253 participants). For both studies, individuals were excluded if they had a history of cerebral vascular accident that precluded the ability to communicate with the researcher.

Regarding the age, in the first study^(^ 14 ^)^, individuals could be included if they were 18 years or older, and in the second study^(^ 2 ^)^ were included 21 years or older individuals. In this present study, the results are regarding all patients were age 18 or older.

Since 25 individuals participated in both studies, we only utilized their data from the earliest evaluation. Therefore, the total number of participants in this analysis was 531. Given the inclusion/exclusion criteria, the final sample can be thought of as representative of individuals hospitalized for cardiac diseases.

Both studies collected the following demographic and clinical data: sex, age in years, marital status (single, widowed, or divorced vs. married or living with significant other), paid job (yes or no), years of education, monthly family income in Reais, diagnosis at hospital admission, and presence of comorbidities (yes or no; arterial hypertension, obesity, dyslipidemia and diabetes mellitus). Diagnosis at hospital admission was categorized into coronary artery diseases (coronary artery disease, angina pectoris, acute myocardial infarction), heart failure (heart failure with or without acute myocardial infarction), or other (heart valve disease, arrhythmia, or other). 

Somatic and cognitive-affective symptoms

Both studies collected information on somatic and cognitive-affective symptoms via the subscales of the BDI-I^(15) ^using the adapted version to Portuguese language^(^ 16 ^)^.

The BDI-I is an inventory composed of 21 items, where each item describes a specific behavioral manifestation of depression. Each item has four choices of self-assessment statements, which are scored 0 to 3, with higher scores indicating increasing symptom severity. Respondents are instructed to describe the way they have been feeling during the past week. Higher sum of scores suggest higher depression symptoms^(^ 15 ^)^. 

Based on one review of existing factor models and item content^(17) ^and studies with cardiac patients^(^ 8 ^,^ 18 ^)^, scores on items 1 to 10 and 12 to 14 (sadness, pessimism, past failure, loss of pleasure, guilty feelings, punishment feelings, self-dislike, self-blame, suicidal thoughts or wishes, crying, withdrawal, indecisiveness, and physical appearance concerns) are summed to calculate the cognitive-affective symptom subscale of the BDI-I (range 0 to 39). Likewise, items 11 and 15 to 21 (irritability, work ability, sleep disturbances, tiredness or fatigue, appetite disturbances, weight disturbances, health worries and sexual disinterest) are summed to calculate the somatic symptom subscale of BDI-I (range 0 to 24). Higher scores in the subscales mean higher somatic and cognitive-affective symptoms.

### Data analysis

Data were analyzed using IBM SPSS version 21.0 for Windows and Mac (SPSS, Inc., Chicago, IL, USA). Descriptive analysis was used for all variables. Demographic and clinical characteristics were compared between males and females by using the Chi-square test (marital status, paid job, presence of arterial hypertension, obesity, dyslipidemia and diabetes mellitus, and diagnosis at hospital admission), or t-test for independent samples (age, monthly family income, and years of education). To compare scores of the subscales of somatic and cognitive-affective symptoms, we used a two-way analysis of variance (ANOVA), using age and sex as factors, and included their interaction. The significance level was set to α=0.05.

### Ethical procedures

This study was approved by the Research Ethics Committee (Comitê de Ética em Pesquisa) of the Hospital das Clínicas of the Faculdade de Medicina de Ribeirão Preto da Universidade de São Paulo (Process HCRP nº. 12164/2012), and it was exempt from a new informed consent, since it was a secondary analysis of data already available from previously approved studies. 

## Results

Table 1 shows the participants' demographic and clinical characteristics. When compared to women, men had slightly higher mean years of education and higher family income (both statistically significant with p<0.001), but were similar in mean age (p=0.30). A higher proportion of men were married or living with a partner (p<0.001) and were employed (p<0.001) than women. 

**Table 1 -:** Distribution of participants by sex according to sociodemographic and clinical characteristics. Ribeirão Preto, SP, Brazil. 2005-2011

Characteristics			Men (n=333)					Women (n=198)				p-value*
			n	%	Mean	Standard deviation		n	%	Mean	Standard deviation	
Sociodemographic characteristics												
	Education in years^†^				5.5	4.3				4.2	3.6	<0.001
	Family income in Reais^†^				1517	1320				939	962	<0.001
	Age in years*				57.3	13.0				56.2	12.1	0.30
	Age group											0.21
		18-44.9	61	18.3				37	18.7			
		45-54.9	69	20.7				51	25.8			
		55-64.9	104	31.2				55	27.8			
		65-74.9	73	21.9				48	24.2			
		75 or older	26	7.8				7	3.5			
	Married/With significant other		253	76.0				113	57.1			<0.001
	Have a paid job		173	52.1				50	25.3			<0.001
Clinical characteristics												
	Diagnosis at admission^‡^											0.005
		Coronary Artery Disease	243	74.1				119	50.7			
		Heart failure	37	11.3				36	18.4			
		Others^‡^	48	14.6				41	20.9			
	Presence of comorbidity											
		Arterial hypertension	219	65.8				151	76.3			0.01
		Obesity^†^	124	37.6				74	37.9			0.93
		Dyslipidemia	114	34.2				106	53.5			<0.001
		Diabetes mellitus	95	28.5				77	38.9			0.01
		Cerebral Vascular Accident	13	3.9				7	3.5			0.83

* p-value from a t-test for education in years, family income, and age in years, and from a Chi-square test for age group, marital status, paid job, diagnosis at hospitalization, and presence of comorbidities

† 1 missing value for education, 11 missing for income, 7 missing for diagnosis at hospital admission, and 6 missing for presence of obesity

‡ Arrhythmias, heart valve disease or Chagas

Men and women differed in diagnosis at hospital admission, with a higher proportion of men having coronary artery disease and higher proportion of women having heart failure. Men had lower proportions of arterial hypertension (p=0.01), dyslipidemia (p<0.001), and diabetes (p=0.01) than women, but were not different in obesity (p=0.93) and cerebral vascular accident (p=0.83). 

Table 2 presents the results of the ANOVA. For both subscales, there was an effect of sex, but no effect of age or the interaction between age and sex. 

**Table 2 -:** P-values from the analysis of variance (ANOVA) for Beck Depression Inventory - I (BDI-I) somatic and cognitive-affective symptoms, according to sex, age group and interaction between sex and age. Ribeirão Preto, SP, Brazil, 2005-2011

Factor	P-value for factor	
	Somatic	Cognitive-affective
Sex	<0.001	0.005
Age group	0.72	0.57
Interaction between Sex and Age	0.26	0.24

Table 3 shows the means and standard deviations for the somatic and cognitive-affective subscales of the BDI separately by sex and age. Women presented with higher values of both somatic and cognitive-affective symptoms than men. 

**Table 3 -:** Means and standard deviations for Beck Depression Inventory - I (BDI-I) somatic and cognitive-affective symptoms, according to total sample, sex and age group. Ribeirão Preto, SP, Brazil, 2005-2011

Characteristic		Somatic			Cognitive-affective	
		Mean	Standard Deviation		Mean	Standard Deviation
Total sample (n=531)		7.3	7.5		6.	4.4
Sex						
	Men	5.4	4.3		6.7	7.2
	Women	7.1	4.5		8.3	7.9
Age group						
	18-44.9	6.4	4.3		7.5	7.8
	45-54.9	6.2	4.4		7.4	7.6
	55-64.9	5.9	4.3		7.6	7.8
	65-74.9	5.8	4.7		6.6	6.8
	75 or older	5.7	4.9		7.3	7.5

**Figure 1 -:**
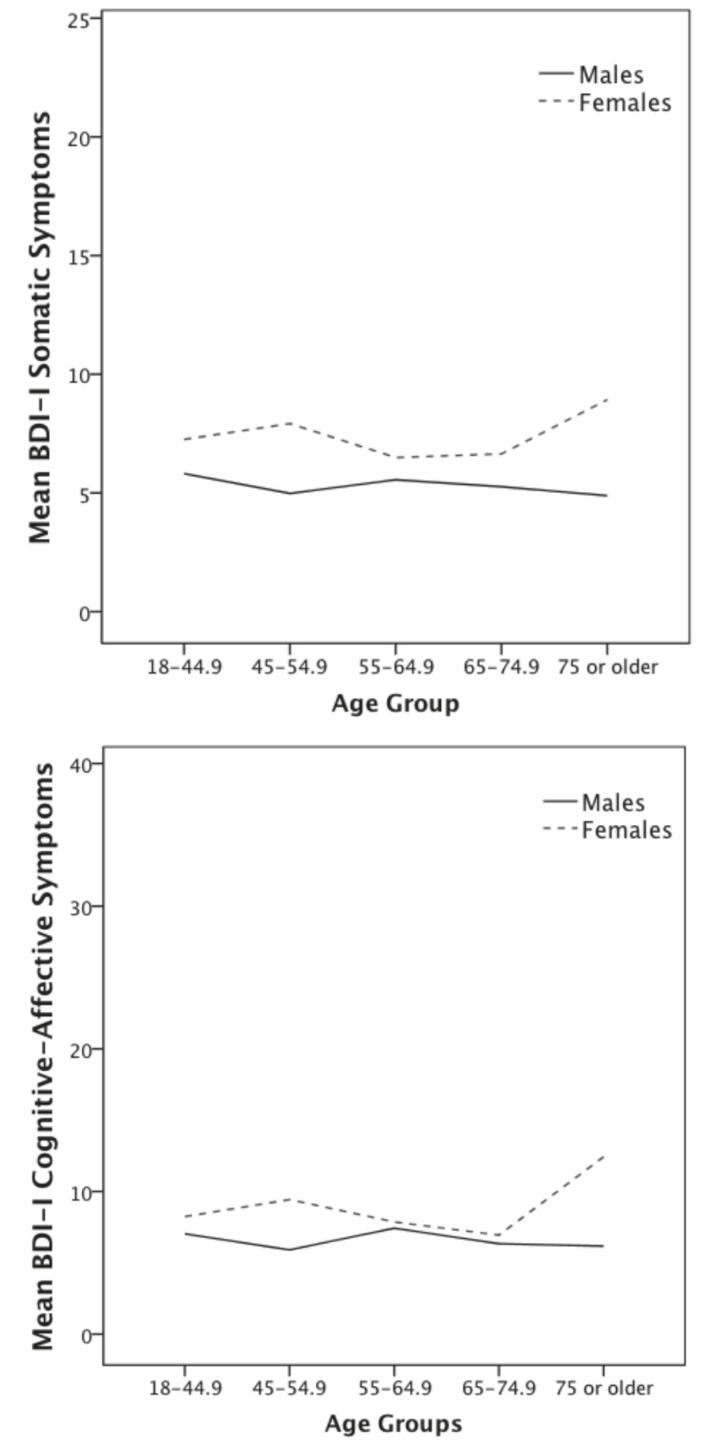
Mean of the BDI-I somatic and cognitiveaffective symptoms by age and sex

Figure 1 depicts the mean of the two BDI-I subscales by age and sex. We plotted the data of each subscale in order to show the relative severity of the symptoms for each age and sex group. Note that males seem to have the same level of symptoms regardless of age group for both, somatic and cognitive-affective subscales. Females presented higher values than males for all age groups, but seemed to have higher values at middle age (45-49.5 years) and older age (75 or more), although age group was not statistically significant.

## Discussion

We found that somatic and cognitive-affective symptoms were different for the groups of men and women, but not different for age groups. 

In the literature, studies that used the BDI-I in populations with cardiac diseases did not report the somatic and cognitive-affective subscales separately and, therefore, a direct comparison with our study is not possible. However, when taken as a single total score, the BDI-I showed differences between males and females in depressive symptoms for various studies^(11,19-21) ^and our general findings are consistent with the literature.

Somatic symptoms have been studied for certain populations using measures other than the BDI. Among patients under treatment for depression, females reported more symptoms of depression overall and somatic complaints when compared to men^(^ 19 ^)^. Somatic complaints that were different between males and females included headache, dizziness, back pain, nausea, muscle pain, hot and cold flashes, and feeling lump on throat. Other researchers also studied people with major depressive disorder (MDD) and found a higher likelihood that females would present with somatic depression than males^(^ 11 ^)^. Fatigue and change in appetite were significantly different between men and women^(^ 11 ^)^. 

In our study, age group was not a statistically significant factor in the presence of cognitive-affective or somatic symptoms. Again, we found no studies that examined cognitive-affective symptoms separately. However, while not statistically significant, we did see a trend where an increase of somatic and cognitive-affective symptoms was seen for those at younger age (especially middle age) when compared to older age. A similar pattern has been observed in the people with depression in the general population^(^ 7 ^,^ 9 ^-^ 10 ^)^. Note in Figure 1 that women had higher means for both subscales of the BDI-I, but there were only seven women in that group and two of them had high-value symptoms, inflating the mean of the entire group. 

Some possible causes of differences in depressive symptoms between men and women have been discussed in the literature^(^ 22 ^-^ 23 ^)^, including a study in the Brazilian population^(^ 24 ^)^. It is thought that depressive symptoms in women might be related to biological aspects, such as hormonal oscillations during their reproductive period and menopause, as well as psychosocial aspects, such as their social and family roles. In the occidental culture, including the Brazilian population, women might have a relatively larger work burden than men, as they might have a professional job while still doing the majority of the housework, being primarily responsible for the care of children, husbands, and family relatives who are sick, all of that while having a lower level of education and receiving lower salaries than men. In our study, we observed a higher percentage of men who were married, having a higher mean family income, and with a higher mean number of years of education at the time of hospitalization. It is possible that these psychosocial factors in addition to their chronic cardiac condition might predispose women to more severe symptoms of depression than men.

Using the subscales of the BDI-I separately for patients with cardiomyopathy has implications for both clinical practice and research. Differentiating the type of depressive symptoms a person has might help in the evaluation of the severity (intensity and chronicity) of the condition, its prognostic, and possible response to treatment, especially when there are differences in the manifestation of depressive symptoms according to the person's characteristics. In our study, women showed higher levels of somatic and cognitive-affective symptoms than men. This fact might be used in the treatment that is prescribed to them. For example, some researchers suggest that person with elevated scores in the cognitive-affective domain might benefit from cognitive behavior treatments, while persons with elevated somatic scores might benefit from medication treatment^(^ 25 ^)^. 

This study had some limitations. First, this is a secondary data analysis and as such, our results are of an exploratory nature. There were variables that were not available and which we feel should be considered in future studies. For example, it would have been interesting to look at whether the hospitalization was the first, second, etc. While one of the studies only had people in their first hospitalization, the other study did not have that information, and therefore, we could not associate that variable with depressive symptoms. 

## Conclusion

Using the subscales of the BDI-I in people with cardiomyopathy sampled from two public hospitals in the state of São Paulo, Brazil, we found that women had, on average, higher levels of somatic and cognitive-affective symptoms than men. Age was not statistically associated with the subscales of BDI-I. Although women in the age group of 45-54.9 years showed a higher mean in both subscales, the interaction of age and sex was not statistically significant. 

Treatment of patients with cardiomyopathy should include the assessment of depressive symptoms and special care should be taken to assess depression in women, so that depressive symptoms are identified early and appropriate treatment is initiated.
